# Association between intoxication with psychoactive substances and adverse effects occurrence in consumers

**DOI:** 10.3389/fpubh.2023.1228854

**Published:** 2023-09-25

**Authors:** Alireza Amanollahi, Shahin Shadnia, Yadollah Mehrabi, Koorosh Etemad

**Affiliations:** ^1^Department of Epidemiology, School of Public Health and Safety, Shahid Beheshti University of Medical Sciences, Tehran, Iran; ^2^Department of Clinical Toxicology, Toxicological Research Center, Loghman Hakim Hospital, School of Medicine, Shahid Beheshti University of Medical Sciences, Tehran, Iran

**Keywords:** intoxication, psychoactive substance, adverse effects, hospitalized, mortality

## Abstract

**Background:**

The prevalence of psychoactive substance use is increasing worldwide and identifying adverse effects of these types of drugs is necessary in intoxicated patients.

**Objective:**

We aimed to investigate the association of psychoactive substance intoxication with their adverse effects on the functioning of the bodily organs.

**Methods:**

This was a single-center study between March 2019 and April 2022 on intoxicated patients with psychoactive substances. Inclusion criteria were intoxication with alcohol, opioids, and stimulants, and having available results of laboratory biomarkers. Demographic and clinical data of patients at the time of admission as well as during hospitalization were reviewed, retrospectively. Data were analyzed using a generalized linear mixed model in R software and the Adjusted Odds Ratio (AOR) was estimated.

**Results:**

A total of 800 hospitalized patients in the ICU (*n* = 400) and general ward (n = 400) were divided into two groups of intoxicated with alcohol (n = 200) and opioids or stimulants (n = 200). Liver (AOR = 0.15, *p* = 0.033; AOR = 0.13, *p* = 0.007) and kidney (AOR = 0.46, *p* = 0.004; AOR = 0.24, *p* = 0.021) dysfunction occurred less in the ICU and general ward, respectively, in opioids or stimulants intoxication compared to alcohol. Cardiovascular dysfunctions occurred more in opioids or stimulants intoxication compared to alcohol in both ICU (AOR = 10.32, *p* < 0.0001) and general ward (AOR = 4.74, *p* < 0.0001).

**Conclusion:**

Kidney dysfunctions had a greater effect on mortality compared to other dysfunctions. During the follow-up, the incidence of dysfunctions increased in those intoxicated with opioids or stimulants. Men experienced more liver and kidney dysfunctions as well as mortality, but psychoactive substance experience was a protective factor in cardiovascular dysfunctions and mortality.

## 1. Introduction

A Psychoactive substance or psychotropic is a form of chemical agent, affecting the function of the human body and a person’s mental and behavioral status. Psychoactive substances are classified into natural, synthetic, and semi-synthetic forms (alcohol, opioids, stimulants, sedative-hypnotics, cannabis, hallucinogens, and inhalants), which differ in influences based on the route of administration (injection, ingestion, smoking, inhalation) and adsorption speed (1, 2). World Drug Report 2020 reported that about 269 million people worldwide had used drugs at least once in 2017 which corresponds to 5.4% of the global population aged 15–64 (range: 3.3 to 7.5%), representing nearly 1 in every 19 people (3). The Middle East/South-West Asia is one of the regions with a high prevalence of opioid use and the prevalence of methamphetamine use in Iran is the highest (3). Acute intoxication is one of the problems that the medical staff working in emergency departments have to deal with, and this department is the first line of treatment for patients who suffer acute intoxication (4).

Irreversible health effects are one of the side effects of opioids and the mortality rate due to opioid overdose in the United States has increased over the years, while the synthesized form of opioids is responsible for most mortality events (5). Results of a cohort study for a 35-year period in Iran have shown that the prevalence of illegal use of drugs is 11.9%, alcohol consumption 9.0%, and co-use of alcohol and illegal drugs 47.7% (6). Another study reported that psychoactive substances including opium, amphetamine, and alcohol are responsible for 30.9% of suicide events and nearly 50% of events are due to addiction (7).

Adverse effects of psychoactive substances on the body depend on the single or multiple drug intoxication, route of administration, and type of combined substance (1, 8). However, the route of drug consumption is varied in different areas owing to specific demographic and socioeconomic characteristics (9). Several side effects of psychoactive substances on organ function have been reported that may differentially contribute to mortality events in terms of the type of consumed drug, history of intoxicated patient (10), and purpose of consumption in patients with drug overdoses (11).

In the present study, we aimed to determine the effects of intoxication with psychoactive substances on the function of the bodily organs including the liver, kidney, and cardiovascular system by investigating related biomarkers in hospitalized intoxicated patients over the course of treatment.

## 2. Methods

### 2.1. Study design and setting

This retrospective longitudinal study was conducted on intoxicated patients with psychoactive substances who were hospitalized either in the general ward or the Intensive Care Unit (ICU) between March 2019 and April 2022 in Loghman Hakim Medical Center (Tehran, Iran). The protocol of the study was approved by the institutional review board and written informed consent was obtained from all patients who participated in the study. Demographic data and medical records of patients were extracted from the Hospital Information System (HIS) and analyzed.

### 2.2. Population and inclusion criteria

Inclusion criteria to enter into the study were monointoxication with alcohol or psychoactive substances including natural, synthetic, and semisynthetic opioids (opium, methadone, cannabis, heroin, and tramadol), and stimulants (amphetamines and cocaine). Also, patients who were hospitalized for 48 h and had repeat testing at least 2 times during hospitalization and age ≥ 16 years were included. Patients who had incomplete medical records or were unwilling to participate in the study were excluded from the study.

### 2.3. Variables and data sources

In order to assess the adverse effects of psychoactive substances on the function of body organs, clinical index related to the function of the liver, kidney, and cardiovascular including aspartate aminotransferase (AST), alanine aminotransferase (ALT), blood urea nitrogen (BUN), creatinine (Cr), troponin, and creatine kinase-MB (CK-MB) were serially checked throughout hospitalization. Moreover, demographic and clinical data including age, gender, purpose (intentional) and history of drug consumption, comorbidities, mean arterial pressure (MAP), Glasgow Coma Scale (GCS), and length of stay (LOS) were extracted from HIS.

### 2.4. Statistical analysis

Data are presented as mean ± standard deviation (SD) or median [IQR] and frequency (%). Normal distribution of data was assessed by the Kolmogorov–Smirnov test, then the student t-test and Mann–Whitney test were applied to compare the mean/median difference between groups. Categorical variables were compared by chi-square or Fisher’s exact test, as appropriate. The generalized linear mixed model (GLMM) estimates fixed and random effects between individual variabilities at baseline and correlation within repeated measures and is especially useful when the dependent variable is binary, ordinal, or quantitative but not normally distributed (12). We used the GLMM model to evaluate the occurrence of adverse effects in intoxicated patients and also effective factors in mortality events in the ICU. Also, the effect of the main covariates including age, gender, purpose, and history of drug consumption along with other covariates were adjusted in the model. We applied Maximum likelihood and Akaik Information Criteria (AIC) for the goodness of fit models and all analyzes were done in R software using the lme4 package.

## 3. Results

### 3.1. Participants and descriptive data

A total of 800 intoxicated patients met our inclusion criteria and were entered into the study. Participants were classified into 2 groups of patients hospitalized in the general ward (n = 400) and ICU (n = 400). Each group of the study consisted of 200 patients with alcohol (methanol) intoxication and 200 patients with opioids or stimulants, which included methadone (142, 17.8%), opium (81, 10.1%), tramadol (63, 7.9%), heroin (17, 2.1%), cannabis (13, 1.6%), amphetamines (67, 8.4%), and cocaine (17, 2.1%). Demographic and clinical data of participants are summarized in Table 1. In the ICU group, intoxicated patients with alcohol had significantly lower Glasgow Coma Scale scores (*p* < 0.001) and a higher level of ALT (*p* = 0.001), AST (*p* = 0.045), Cr (p = 0.001) while CK-MB level was significantly lower (p = 0.001). Positive troponin test (>12 ng/L) among patients with opioid or stimulant intoxication was remarkably more common in both ICU (*p* = 0.003) and general ward (*p* = 0.036) when compared with alcohol intoxication. Also, the past medical history of patients revealed that psychoactive consumption (*p* = 0.009) and liver disease (*p* = 0.019) differed significantly between the two groups of ICU patients. Regarding patients in the general ward, intoxication with alcohol was notably more common in men (*p* = 0.033) and levels of ALT (*p* = 0.009) and Cr (*p* = 0.001) were significantly higher whereas PH value was lower (p = 0.001) when compared with opioids or stimulants intoxication.

**Table 1:** Basic demographic and clinical characteristics of intoxicated patients.

Variables	ICU (*n* = 400)			General ward (*n* = 400)		
	Opioids or Stimulants	Alcohol	*p* value	Opioids or Stimulants	Alcohol	*p*-value
Age (year)	40.31 ± 18.71	38.70 ± 14.38	0.335	38.99 ± 17.21	36.13 ± 14.16	0.071
Sex (Male)^a^	163 (81.5)	163 (81.5)	0.999	157 (79.0)	174 (87.0)	0.033
LOS (days)	7.37 ± 6.58	7.54 ± 6.75	0.977	1.80 ± 1.31	1.85 ± 1.33	0.422
MAP (mmHg)	87.93 ± 15.18	89.56 ± 17.97	0.324	89.78 ± 14.08	89.07 ± 13.65	0.611
GCS	10.38 ± 4.10	8.24 ± 4.08	<0.001	12.92 ± 3.15	13.97 ± 2.35	0.110
Psychoactive use (yes)	113 (56.5)	87 (43.5)	0.009	97 (48.5)	89 (44.5)	0.423
Intentional use (yes)	164 (82.0)	151 (75.5)	0.112	169 (84.5)	171 (85.5)	0.779
Liver disease (yes)	2 (1.0)	10 (5.0)	0.019	3 (1.5)	4 (2.0)	0.997
Kidney disease (yes)	5 (2.5)	5 (2.5)	0.999	8 (4.0)	4 (2.0)	0.241
Cardiovascular disease (yes)	21 (10.5)	24 (12.0)	0.635	13 (6.5)	10 (5.0)	0.519
ALT (up to 41 U/L)^b^	38 (21–108)	51 (27–101.5)	0.001	30 (18–63)	38 (21–75)	0.009
AST (up to 43 U/L)^b^	52 (33–106)	60 (35–119)	0.045	38 (21–75)	47 (30.5–88)	0.524
BUN (0.6–1.3 mg/dL)^b^	35 (22–73)	38 (25–58)	0.222	32 (24–44)	35 (25–47)	0.236
Cr (< 1.4 U/L)^b^	1.1 (0.9–1.7)	1.2 (0.9–1.7)	<0.001	1.1 (0.95–1.3)	1.3 (1.1–1.6)	<0.001
CK-MB (<24 U/L)^b^	65 (25–255)	36 (19–85)	0.001	36 (22–80)	27 (15–53)	0.106
CPK (24–195 U/L)^b^	501.5 (180.5–2052)	525 (173.5–2,118)	0.724	412 (159.5–1979)	303 (139.5–988.5)	0.017
PH (7.35–7.45)^b^	7.346 (7.282–7.403)	7.348 (7.246–7.426)	0.891	7.372 (7.332–7.416)	7.353 (7.262–7.425)	<0.001
Troponin I (>12)	52 (26)	28 (14)	0.003	28 (14)	15 (7.5)	0.036
Mortality	51 (42.9)	68 (57.1)	0.063	–	14 (100)	–

a Categorical data as *N* (%).

b Continues data as Median (IQR), Mean ± SD.

LOS, length of stay, MAP, mean arterial pressure, GCS, Glasgow coma scale, ALT, Alanine aminotransferase, AST, Aspartate aminotransferase, BUN, Blood Urea Nitrogen, Cr, Creatinine, CK-MB, Creatine Kinase-MB, CPK, Creatine Phosphokinase.

### 3.2. Outcome data

**Figure 1:**
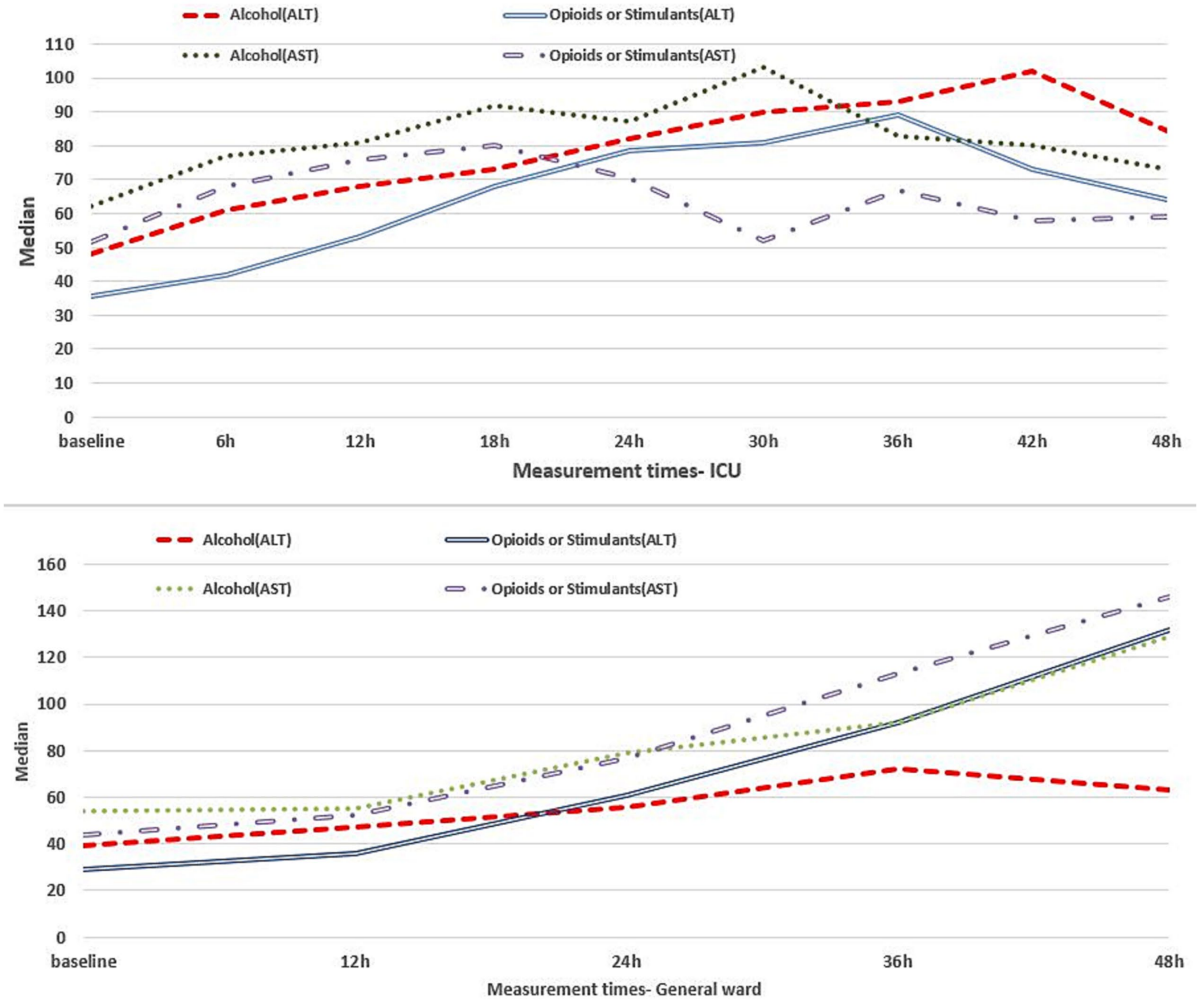
Liver clinical indexes for intoxicated patients in the ICU (upper panel) and general ward (lower panel) within 48 h of hospitalization.

**Figure 2:**
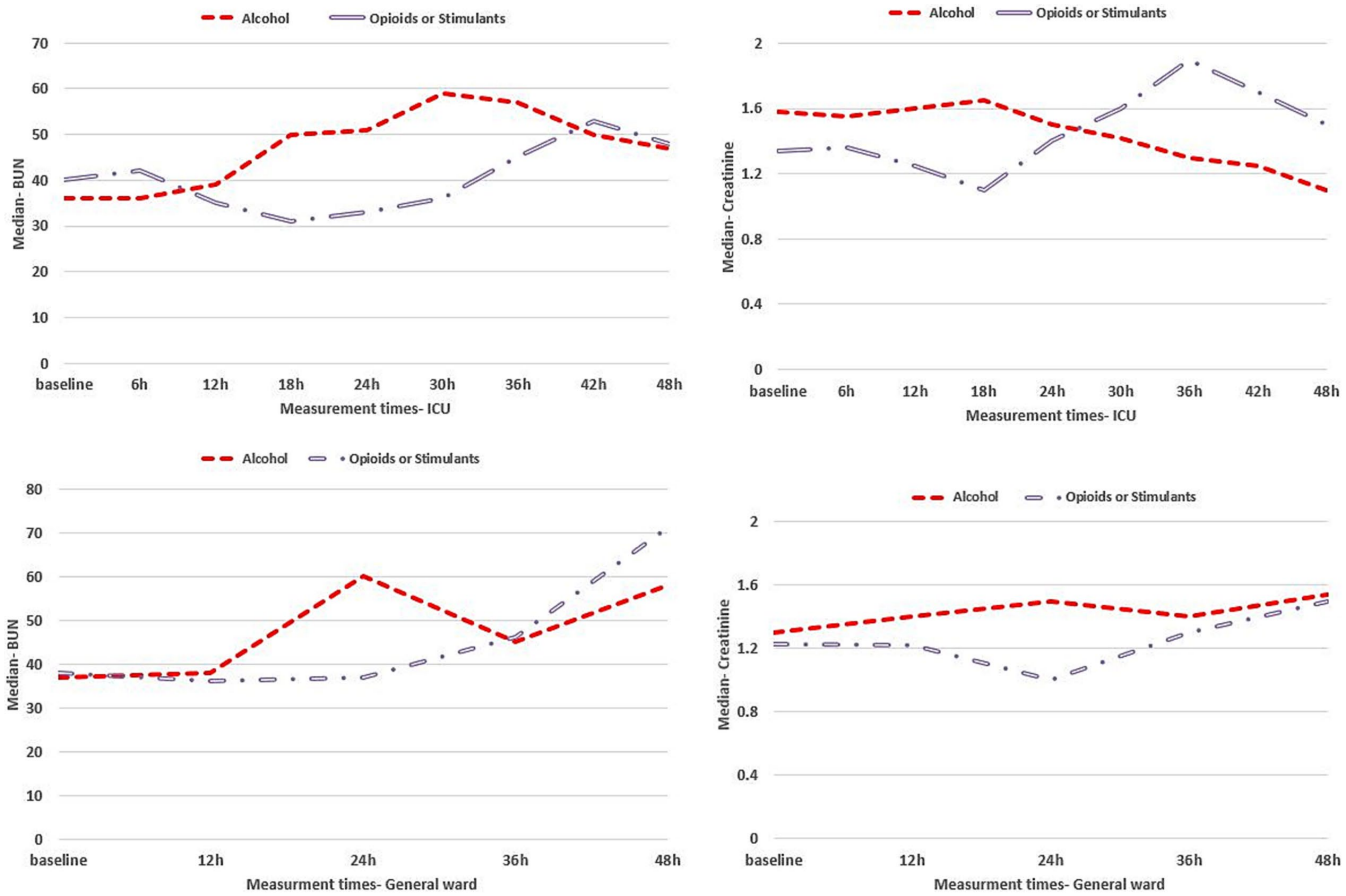
Kidney clinical indexes for intoxicated patients in the ICU (upper panels) and general ward (lower panels) within 48 h of hospitalization.

**Figure 3:**
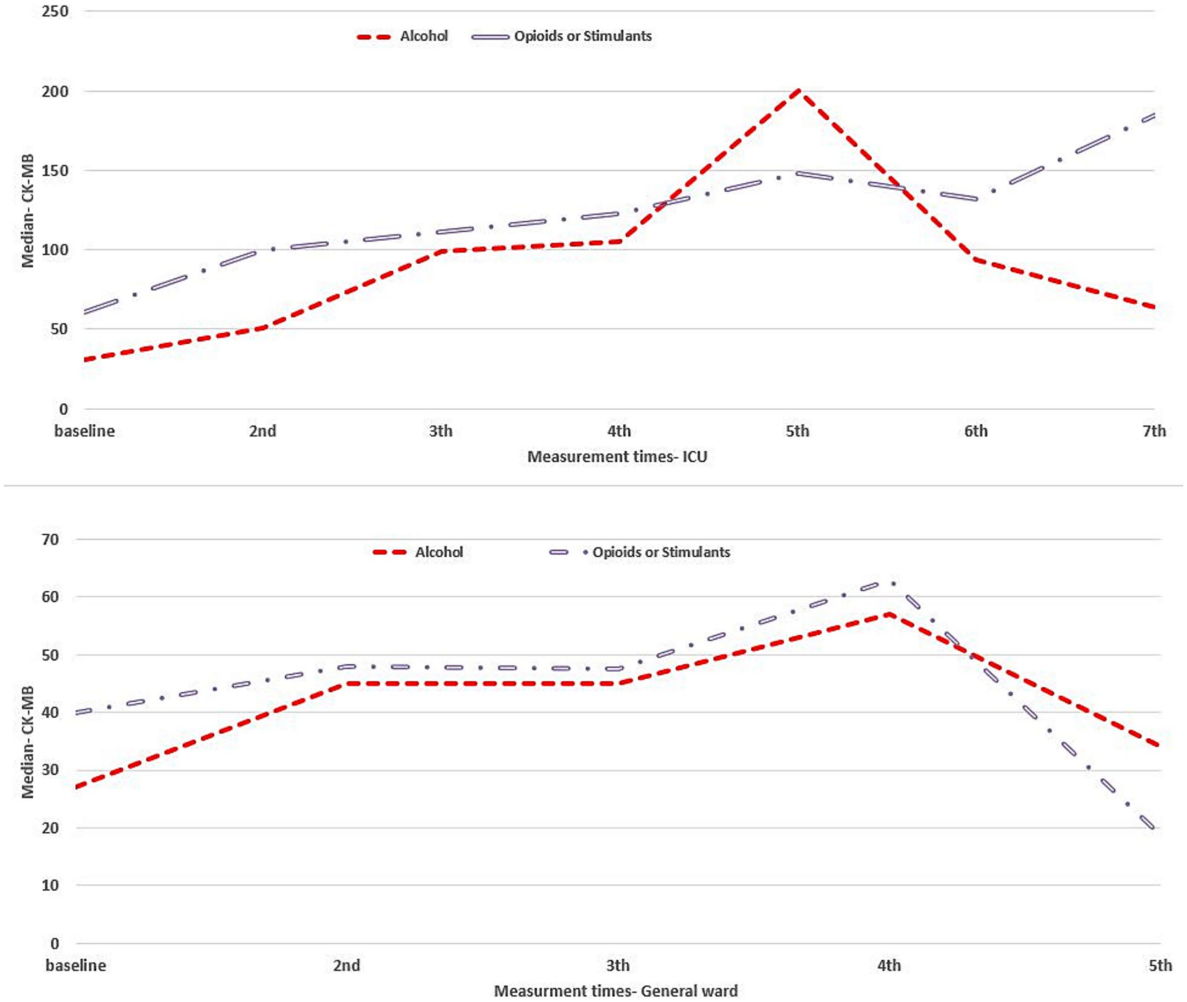
Cardiovascular clinical index for intoxicated patients in the ICU (upper panel) and general ward (lower panel) duration of hospitalization.

Observed adverse effects in patients with alcohol intoxication who were hospitalized in ICU and general ward were liver dysfunction (81% vs. 58%), kidney dysfunction (83.5% vs. 52%), and cardiovascular dysfunction (14% vs. 7.5%), respectively, while adverse effects in opioid or stimulant intoxicated patients in ICU and general ward, respectively, were liver dysfunction (70% vs. 42%), kidney dysfunction (70.5% vs. 34%), and cardiovascular dysfunction (26% vs. 14%). Mortality event was observed in 119 (28.9%) ICU patients, of which 57.1% were alcohol intoxicated and 42.9% were opioid or stimulant intoxicated. Longitudinal changes in clinical indexes related to body organ function are represented in Figure 1 for the liver, Figure 2 for the kidney, and Figure 3 for the cardiovascular within 48 h of hospitalization.

### 3.3. Main results

Table 2 shows the association between the occurrence of dysfunctions (liver, kidney, and cardiovascular) in intoxicated patients with opioids or stimulants compared to alcohol during hospitalization. Adjusted Odds Ratio (AOR) of the dysfunctions at baseline is compared between the two groups. AOR for liver and kidney dysfunctions was significantly lower in the opioid or stimulant intoxication group in both the ICU and in general wards. Accordingly, AOR for liver dysfunction in ICU was 0.15 (*p* = 0.033), and for kidney dysfunction was 0.46 (*p* = 0.004). In the general ward, the estimated AOR was 0.13 (*p* = 0.007) for the liver and 0.24 (*p* = 0.021) for kidney dysfunction. Regarding cardiovascular dysfunction, AOR was notably higher in patients with opioid or stimulant intoxication compared to the alcohol group. The estimated AOR for cardiovascular dysfunction occurrence in the ICU and general ward, respectively, was 10.32 (*p* < 0.0001) and 4.74 (*p* < 0.0001). Over the course of hospitalization, the occurrence of liver and kidney dysfunction in the opioids or stimulants group increased in a time-dependent manner, whereas cardiovascular dysfunction occurred more in the alcohol group. Furthermore, AOR for the occurrence of dysfunction increased with the increase of the patient’s age. Among the ICU patients, AOR of liver and kidney dysfunction was higher in men and cardiovascular dysfunction was more frequent in women compared to men in both ICU and general ward. Also, a history of psychoactive substance use was a risk factor for the occurrence of liver and kidney dysfunction and a protective factor for cardiovascular dysfunction. The association between mortality and organ dysfunctions in ICU patients is shown in Table 3. Kidney dysfunction had a significantly high AOR of 3.35 (*p* = 0.0002) for mortality in hospitalized patients. The AOR of mortality also increased with the increase of age and duration of hospitalization and decrease of MAP and GCS.

**Table 2:** Occurrence of organ dysfunctions in opioids or stimulants versus alcohol intoxication.

Ward	Variables	Liver		Kidney		Cardiovascular	
		AOR^c^ (%95 CI)	*p*-value	AOR^c^ (%95 CI)	*p*-value	AOR^c^ (%95 CI)	*p*-value
ICU	Group (Opioid or Stimulant)^a^	0.15 (0.03–0.86)	0.033	0.46 (0.27–0.78)	0.004	10.32 (10.27–10.36)	<0.0001
	Time	1.67 (1.57–1.77)	<0.0001	1.05(1.02–1.08)	0.001	0.844 (0.841–0.846)	<0.0001
	Age (year)	1.03 (1.02–1.04)	<0.0001	1.02 (1.009–1.04)	0.001	1.05 (1.04–1.06)	<0.0001
	Sex (M)	3.20 (3.11–3.29)	<0.0001	1.72 (0.95–3.13)	0.072	0.997 (0.994–1.001)	0.156
	Intentional use (yes)	1.17 (1.09–1.25)	<0.0001	2.09 (1.15–3.78)	0.015	1.13 (1.12–1.14)	<0.0001
	History of psychoactive use (yes)	1.15 (1.13–1.17)	<0.0001	1.13 (0.70–1.83)	0.608	0.46 (0.45–0.47)	<0.0001
	Group^*^time^b^	1.02 (1.01–1.03)	<0.0001	1.009 (0.97–1.05)	0.651	1.07 (1.05–1.09)	<0.0001
General	Group (Opioid or Stimulant) ^a^	0.13 (0.03–0.52)	0.007	0.24 (0.07–0.81)	0.021	4.74 (4.73–4.75)	<0.0001
	Time^b^	0.76 (0.56–1.03)	0.077	1.22 (0.93–1.59)	0.141	0.21 (0.20–0.22)	<0.0001
	Age (year)	0.98 (0.95–1.02)	0.463	1.08 (1.05–1.12)	<0.0001	1.03 (1.02–1.04)	<0.0001
	Sex (M)	3.96 (0.88–17.83)	0.072	0.97 (0.31–3.13)	0.970	0.25 (0.23–0.27)	<0.0001
	Intentional use (yes)	0.28 (0.05–1.48)	0.135	1.42 (0.43–4.71)	0.568	2.96 (2.91–3.01)	<0.0001
	History of psychoactive use (yes)	1.59 (0.48–5.21)	0.443	2.16 (0.87–5.38)	0.093	0.44 (0.42–0.46)	<0.0001
	Group^*^time^b^	1.34 (0.85–2.12)	0.201	1.06 (0.70–1.61)	0.782	0.50 (0.48–0.52)	<0.0001

a Group: opioid or stimulant vs. alcohol intoxication at the baseline.

b Interaction between group with time: changes in occurrence of dysfunctions during hospitalization.

c Adjusted odds ratio for length of stay, mean arterial pressure, Glasgow Coma Scale.

**Table 3:** Association between patient variables and mortality in ICU.

Variables	AOR (%95 CI)	*p*-value
Opioid or Stimulant	0.37 (0.42–1.27)	0.263
Liver dysfunction (yes)	1.94 (1.11–3.39)	0.019
Kidney dysfunction (yes)	3.35 (1.76–6.37)	0.0002
Cardiovascular dysfunction (yes)	1.45 (0.52–4.03)	0.479
Age (year)	1.04 (1.02–1.06)	0.0001
Sex (M)	1.64 (0.80–3.37)	0.174
Intentional use (yes)	0.97 (0.52–1.83)	0.928
History of psychoactive use (yes)	0.82 (0.48–1.39)	0.465
LOS (days)	1.04 (1.003–1.08)	0.037
MAP (mmHg)	0.98 (0.96–0.99)	0.010
GCS	0.93 (0.87–0.96)	0.036

## 4. Discussion

### 4.1. Principal finding

Our findings in this study showed that the occurrence of liver and kidney dysfunction was more common in intoxication with alcohol when compared with opioids or stimulants while the incidence of cardiovascular dysfunctions was higher in opioids or stimulants compared with alcohol. Moreover, mortality was higher in patients with alcohol intoxication in the ICU. Among the evaluated dysfunctions, kidney dysfunction showed the largest effect size on mortality events.

Diagnosis of liver dysfunction is possible through assessing serum levels of aminotransferases and hyperbilirubinemia as laboratory biomarkers of liver function (13). We observed a higher level of aminotransferases in the alcohol intoxication group compared to another group of patients who were intoxicated with psychoactive substances. In addition, pancreatitis and acute kidney injury (AKI) were reported in alcohol intoxication, especially with methanol, which is mostly due to myoglobinuria (14, 15). However, the symptoms of alcohol intoxication depend on the level of alcohol in the person’s body, tolerance to alcohol, weight, and the percentage of consumed alcohol (16). Mechanistically, oxidative stress and mitochondrial dysfunction are the main mechanisms contributing to liver dysfunction following alcohol intoxication (17).

In the laboratory setting, liver enzymes and kidney function biomarkers are utilized to diagnose hepatotoxicity and AKI in chronic alcohol consumption. Urinary biomarkers can also be used to follow up clinical condition of patients with alcohol intoxication (18) serial checking of biomarkers improves the patient management process by regular monitoring of hepatorenal function in those patients who develop hepatotoxicity and nephrotoxicity. In our under study population, early liver and kidney dysfunction occurred more in alcohol-intoxicated patients but late hepatorenal dysfunction was observed more during hospitalization in intoxicated patients with opioids or stimulants. Clinical outcomes may be affected by some factors including the half-life of substances, time from consumption to referring hospital, type of admission and speed of absorption, illegal handmade or commercial, quality of pre-hospital care, and undetectable components of illegal drugs. Metabolic acidosis and raised plasma osmolality are more common in alcohol intoxication but rhabdomyolysis, AKI, hypothermia, coma, and pulmonary edema are frequent in alcohol or opioid-intoxicated patients (19).

Cardiovascular dysfunction is more common in opioid intoxication compared to alcohol or stimulants. This phenomenon is mostly due to different toxidrome between opioids, stimulants, and alcohol (16, 19). Cardiotoxicity (20) or cardiomyopathy (21) are the most important contributing factors in the mortality of patients with amphetamines or cocaine intoxication, identified by pathological or electrocardiograph parameters. In addition, duration of consumption is an effective parameter in deteriorating a patient’s cardiovascular condition over hospitalization (22). The differences in the compounds of substances particularly synthesized forms, lead to uncertainty in generalizing obtained results. Furthermore, the composition of a particular substance may be different in a neighborhood, city, country, or continent which ultimately affects the severity and occurrence of side effects. Hence, we tried to reduce these errors in our study by selecting monointoxicated patients in the hospital. The association between the occurrence of cardiac arrest and opioid overdose has been confirmed in the pre-hospital period (23) and over the hospitalization period (24), resulting from cardiac toxicity and respiratory arrest following opioid overdose. In addition to this, attention should be paid to AKI which occurs in amphetamines or opioid-intoxicated patients resulting in rhabdomyolysis, high serum myoglobin, and ultimately nephropathy in consumers (25).

Another concern that should be considered is the increase in the possibility of intoxication due to the prescription of opioids in the hospital, especially in the ICU (26), and in methadone maintenance treatment which is used for Opioid Use Disorder (OUD) (27). Therefore, it is important to closely monitor patients’ clinical conditions and protocol for opioid administration. In recent years, mortality and hospitalizing rates have increased due to opioid overdose (28). Compared to ethanol, the odds ratio of mortality is higher in opioids and stimulants (29). We observed dissimilar results in our studied patients in the ICU and mortality also was more in alcohol overdose compared to opioids or stimulants. The median of laboratory results is reported to be high in methanol intoxications with unfavorable outcomes and shows a significant association with the number of hemodialysis sessions and Sequential Organ Failure Assessment (SOFA) score (30). Age, GCS, and MAP in methanol intoxication are the main risk factors for mortality in comparison with other sequelae (31, 32). In our study, age, MAP, and GCS showed a significant association with mortality in all comparisons. Male gender, intentional use, and no history of substance use were linked to mortality in overdoses with stimulants compared to opioids.

Conflicting results have been reported for the intentional use of substances and side effects. Although 82.2% of patients with AKI had unintentional exposure, the level of ethanol and methanol was higher in patients without kidney injury. Unintentional use of substances may be the reason for not referring to the hospital quickly or not receiving medical services. This claim is documented by the high average time from exposure to arriving at the hospital or even receiving the initiation of hemodialysis (15).

Global health communities should inform people about the adverse effects of consuming any type of psychoactive substances and also be aware of the severe side effects of substances for consumers. In the case of an overdose or change in clinical conditions of consumers, they should seek medical centers, immediately. In order to achieve effective care services, patients should have proper cooperation with health care providers in the physical exam, type of consumed substance, and time interval from consumption to arrival at the hospital. Novel synthetic opioids and substances made by clandestine laboratories are not detectable by conventional techniques, therefore, healthcare system labs need to be updated (33).

### 4.2. Limitations

Dissimilarity of the history of type psychoactive substances used by individuals, the inability of the hospital laboratory to detect some substances, and receiving different doses and types of opioids during the hospitalization are limitations of the study that may affect the results.

## 5. Conclusion

Although the incidence of liver and kidney dysfunction was more common in alcohol intoxication, patients with opioid or stimulant intoxication experienced liver and kidney dysfunction later over the course of hospitalization. Also, the incidence of cardiovascular dysfunction was higher in opioids or stimulant intoxication compared to alcohol. The occurrence of all dysfunctions was more frequent in older patients and females showed cardiovascular dysfunction more. In patients with a history of psychoactive substance use, liver dysfunction was observed more whereas cardiovascular dysfunction was less.

## Data availability statement

The raw data supporting the conclusions of this article will be made available by the authors, without undue reservation.

## Ethics statement

The studies involving human participants were reviewed and approved by Ethical approval has been confirmed by the Research Ethics Committees of the Shahid Beheshti University of Medical Sciences. IR.SBMU.PHNS.REC.1400.162. The patients/participants provided their written informed consent to participate in this study.

## Author contributions

AA: conceptualization, methodology, formal analysis, investigation, visualization, and writing – original draft. SS: conceptualization, resources, visualization, and writing – review and editing. YM: conceptualization, methodology, validation, data curation, visualization, and writing – original draft. KE: conceptualization, methodology, data curation, visualization, writing – review and editing, supervision, and project administration. All authors contributed to the article and approved the submitted version.

## Funding

This research was financially supported by Shahid Beheshti University of Medical Sciences. The funders had no role in the publishing process. KE received a grant for data collection and analysis.

## Conflict of interest

The authors declare that the research was conducted in the absence of any commercial or financial relationships that could be construed as a potential conflict of interest.

## Publisher’s note

All claims expressed in this article are solely those of the authors and do not necessarily represent those of their affiliated organizations, or those of the publisher, the editors and the reviewers. Any product that may be evaluated in this article, or claim that may be made by its manufacturer, is not guaranteed or endorsed by the publisher.
